# Meta-Analysis: Randomized Trials of *Lactobacillus plantarum* on Immune Regulation Over the Last Decades

**DOI:** 10.3389/fimmu.2021.643420

**Published:** 2021-03-22

**Authors:** Wei Zhao, Chuantao Peng, Hafiz Arbab Sakandar, Lai-Yu Kwok, Wenyi Zhang

**Affiliations:** ^1^Key Laboratory of Dairy Biotechnology and Engineering, Ministry of Education, Inner Mongolia Agricultural University, Hohhot, China; ^2^Key Laboratory of Dairy Products Processing, Ministry of Agriculture and Rural Affairs, Inner Mongolia Agricultural University, Hohhot, China; ^3^Inner Mongolia Key Laboratory of Dairy Biotechnology and Engineering, Inner Mongolia Agricultural University, Hohhot, China; ^4^Qingdao Special Food Research Institute, Qingdao Agricultural University, Qingdao, China

**Keywords:** *Lactobacillus plantarum*, immunity, meta-analysis, IL-4, IL-10, TNF-α, IFN-γ

## Abstract

*Lactobacillus* (*L*.) *plantarum* strains, belong to lactic acid bacteria group, are considered indispensable probiotics. Here, we performed meta-analysis to evaluate the regulatory effects of *L. plantarum* on the immunity during clinical trials. This meta-analysis was conducted by searching across four most common literature databases, namely, Cochrane Central Register of Controlled Trials, Web of Science, Embase, and PubMed. Clinical trial articles that met the inclusion and exclusion criteria were analyzed by Review Manager (version 5.3). *p*-value < 0.05 of the total effect was considered statistically significant. Finally, total of 677 references were retrieved, among which six references and 18 randomized controlled trials were included in the meta-analysis. The mean differences observed at 95% confidence interval: interleukin (IL)-4, −0.48 pg/mL (−0.79 to −0.17; *p* < 0.05); IL-10, 9.88 pg/mL (6.52 to 13.2; *p* < 0.05); tumor necrosis factor (TNF)-α, −2.34 pg/mL (−3.5 to −1.19; *p* < 0.05); interferon (IFN)-γ, −0.99 pg/mL (−1.56 to −0.41; *p* < 0.05). Therefore, meta-analysis results suggested that *L. plantarum* could promote host immunity by regulating pro-inflammatory and anti-inflammatory cytokines.

## Introduction

Probiotics have been studied extensively by researchers since their discovery in early twentieth century by Elie Metchnikoff. Their probiotic effects are well-acclaimed by researchers and consumers. Probiotics regulate the gut microbiome, maintain intestinal homeostasis, and modulate other physiological conditions (1). Probiotic products are used to treat/alleviate clinical conditions like constipation, cardiovascular diseases, hyperlipidemia, hyperglycemia, and hypertension (2, 3). Lactobacilli are commonly found in fermented food products and are frequently used in industrial food fermentation (4), and many members of this genera are generally recognized as safe (GRAS). Lactobacilli are Gram-positive bacteria which are found in various natural environments. Modern food and health industries have been investigating and developing a broad range of probiotic strains which have been isolated from nutrient rich habitats such foods, feeds, plants, animals, and humans. It has been substantially reported that probiotics confer beneficial effects to human health (5). Recent works have also shown that the administration of lactobacilli have abated antibiotic-induced side effects, such as gut dysbiosis, diarrhea, and immune dysregulation (6–8). Some of the most used probiotic *Lactobacillus* spp. are *Lactobacillus acidophilus, Lactobacillus casei*, and *Lactobacillus plantarum* (*L. plantarum*) among others.

Recently, *L. plantarum* has been applied in health-promoting products as some strains have shown promising clinical results, such as regulating gastrointestinal function, lowering serum cholesterol, and enhancing immunity (9–11). One of the most obvious beneficial health effects of consuming probiotic products is immune regulation. With the advent in cell biology and molecular biology, a deeper understanding of how probiotics modulate the immune system has been gained (12, 13). Robust evidence has come from clinical and experimental works on the *L. plantarum* species. This species has been found to regulate the innate and adapted cellular and humoral immunity (14). For example, Prakoeswa and his coworkers (15) had shown that taking *L. plantarum* IS-10506 could boost the levels of interleukin (IL)-4 and IL-17 of adults with atopic dermatitis. Moreover, *L. plantarum* could alleviate oxidative stress after a triathlon by increasing the levels of anti-inflammation (IL-10) while decreasing the level of pro-inflammation [tumor necrosis factor (TNF)-α, IL-6, and IL-8] (16). Besides, *L. plantarum* IS-10506 could regulate the immune system by decreasing IL-17, IL-4, and interferon (IFN)-γ levels in serum of children with atopic dermatitis (17).

Meta-analysis was first christened by Glass, a British psychologist (18). In 2001, Egger and colleagues (19) made point that meta-analysis is a valid method to evaluate the effects of the interventions on subjects by combining the results of multiple research data for statistical analysis. Some researchers have used meta-analysis to explore the therapeutic effects of probiotics on allergies and the modulation of intestinal flora (20, 21). *L. plantarum* has many physiological functions, among which immunity is one of the most important (11). However, to the best of our knowledge, there is no specific data indicating the effects of *L. plantarum* in immunity regulation. Therefore, we designed this study to perform meta-analysis on effects of *L. plantarum* administration, mainly focusing on the immunoregulatory studies conducted in the last decades.

## Methods

### Study Strategy

Four databases, namely Cochrane Central Register of Controlled Trials (https://www-cochranelibrary-com.ezproxy.cul.columbia.edu/), Web of Science (http://apps.webofknowledge.com/), Embase (https://www-embase-com.ezproxy.cul.columbia.edu/#search), and PubMed (https://www.ncbi.nlm.nih.gov/pubmed/), were selected for reference retrieval. All the studies on the effect of intervention of *L. plantarum* on host immune published in and before October 2019 were included. The “subject” field (including title, keyword and abstract) was used to retrieve literature. The combination of keywords was based on Boolean operation, and the use of keywords was as follows: (*Lactobacillus plantarum* or *L. plantarum*) and (immunological/immunity/immune/allergy/eczema/rhinitis/asthma/cytokines/atopic dermatitis).

Exclusion criteria: (1) meta-analysis, letter, review, report, meeting summary, etc.; (2) animal test, *in vitro* test, and other non-clinical trials; (3) studies without original data; (4) studies applying prebiotics or other additives; (5) non-randomized controlled trials.

Inclusion criteria: (1) clinical studies of *L. plantarum* taken by human subjects; (2) studies employing immunity-related outcome indicators; (3) randomized controlled trials.

### Data Collection

The data were extracted from the articles that met the inclusion and exclusion criteria. More than two people independently extracted the data, made decisions, and compared the conclusions of each article. In case of any disagreement, a third individual member was introduced to solve the problem through discussion and consultation. When extracting the required data from the original research, the authenticity and reliability of the data were considered. Bias caused by the subjective judgment of the evaluator was avoided as much as possible. If the discussion still failed to solve the existing problems, the details of problems occurring in the data extraction process were to be included. To ensure the reliability of the extracted data and minimize bias and errors, systematic training was given to the members who participated in the analysis and data extraction.

The information extracted in this study included: type of trial, probiotic strains, number of participants, type of intervention, and intervention results [tumor necrosis factor (TNF)-α, IL-4, IL-10, and IFN-γ].

### Data Organization and Analysis

Before the meta-analysis, the units of indicators were standardized. Review Manager 5.3 (Cochrane Collaboration Network, London, UK) was used to conduct this meta-analysis. Forest plots were used to detect the heterogeneity of the articles. Funnel maps were used to detect the possible publication bias (22). The sample size, mean difference, and 95% confidence interval (CI) were calculated to analyze the results of continuous variables. The average difference was calculated by subtracting the baseline data from the data after intervention, and the difference was directly used if given in the original article. If 95% CI includes zero, there is no statistical significance. If the data after intervention and the baseline data were given, statistical analysis was done after calculation. *p*-value < 0.05 of the total effect quantity was considered statistically significant in the confidence interval method. I^2^ was an indicator that measured the degree of heterogeneity in multiple studies. If I^2^ was <50%, the heterogeneity between studies was considered acceptable. When there was no heterogeneity between studies, the fixed effect model method should be used; otherwise, the random effect model should be applied (23).

## Results

### Results of Literature Search

**Figure 1:**
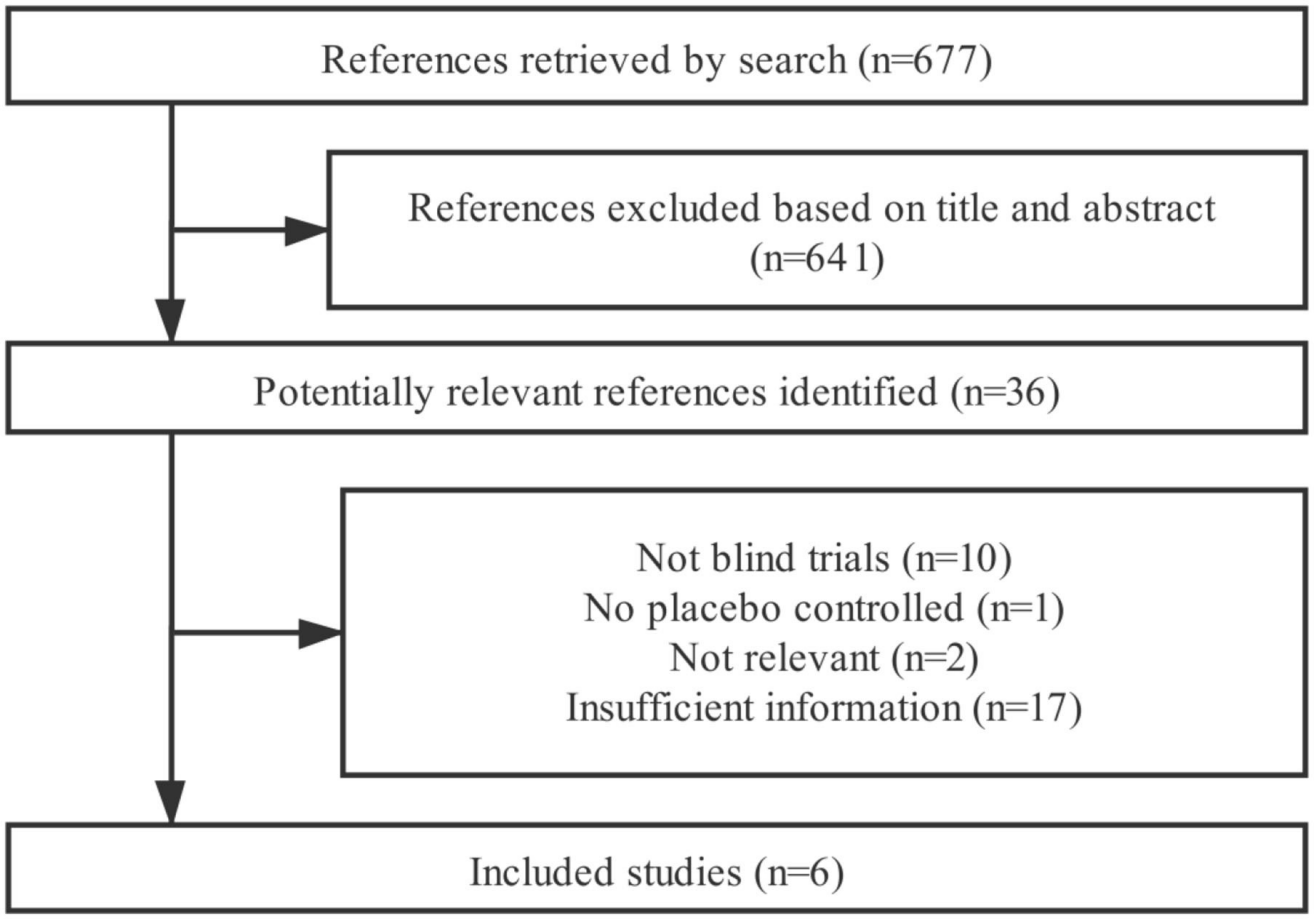
Flow chart of the meta-analysis.

In total, 677 references were retrieved from the four databases (after removing duplicated studies) (Figure 1). Based on the title and abstract, 641 irrelevant articles were excluded since those were animal trials and *in vitro* studies. The remaining 36 articles were relevant after reading the full text. Thirty of these articles were excluded according to the inclusion and exclusion criteria (two articles were not related to this meta-analysis, one article was not placebo-controlled trial, 17 articles were insufficient, and ten articles were based on non-blind trials). Eventually, only six articles were considered in this current meta-analysis. Among the included articles: Costabile et al. (24) measured two immune-related indicators, so this study was considered as two independent trials; Hirose et al. (25) measured two different immune-related indicators; Huang et al. (26) measured four different immune-related indicators; Lew et al. (27) measured four different immune-related indicators; Prakoeswa et al. (17) measured three different immune-related indicators; and Yang et al. (28) measured three different immune-related indicators. Therefore, this meta-analysis included 18 randomized controlled trials.

### Results of Data Extraction

Data were extracted from the selected clinical trials. Extracted data, including probiotic strains, interventions, experiment types, intervention doses, sample size, and outcome index information, are presented in Table 1. The total number of participants was 1,047, including 514 in the probiotic group and 533 in the placebo group. Moreover, the immune parameters selected for this meta-analysis were TNF-α, IL-4, IFN-γ, and IL-10.

**Table 1:** Extracted information.

***Lactobacillus plantarum* strain**	**Form of supplement**	**Dose (CFU/d)**	**Sample size (Probioic/Placebo)**	**Duration (weeks)**	**Outcome indicator (pg/mL)**	**Trial**
L-137	Tablet	2 × 10^9^	15/20	12	IL-4	(25)
PS128	Capsule	3 × 10^10^	24/25	8	IL-4	(26)
P-8	Powder	2 × 10^10^	51/52	12	IL-4	(27)
IS-10506	Microencapsule	1.29 × 10^12^	12/10	12	IL-4	(17)
Not specified	Powder	2 × 10^9^	34/37	6	IL-4	(28)
ECGC 13110402	Capsule	2 × 10^9^	23/22	12	IL-10	(24)
PS128	Capsule	3 × 10^10^	24/25	8	IL-10	(26)
P-8	Powder	2 × 10^10^	51/52	12	IL-10	(27)
IS-10506	Microencapsule	1.29 × 10^12^	12/10	12	IL-10	(17)
Not specified	Powder	2 × 10^9^	34/37	6	IL-10	(28)
ECGC 13110402	Capsule	2 × 10^9^	23/22	12	TNF-α	(24)
PS128	Capsule	3 × 10^10^	24/25	8	TNF-α	(26)
P-8	Capsule	2 × 10^10^	51/52	12	TNF-α	(27)
Not specified	Powder	2 × 10^9^	34/37	6	TNF-α	(28)
L-137	Tablet	2 × 10^9^	15/20	12	IFN-γ	(25)
PS128	Capsule	3 × 10^10^	24/25	8	IFN-γ	(26)
P-8	Capsule	2 × 10^10^	51/52	12	IFN-γ	(27)
IS-10506	Microencapsule	1.29 × 10^12^	12/10	12	IFN-γ	(17)

*All studies were parallel clinical trials*.

### Data Analysis

**Figure 2:**
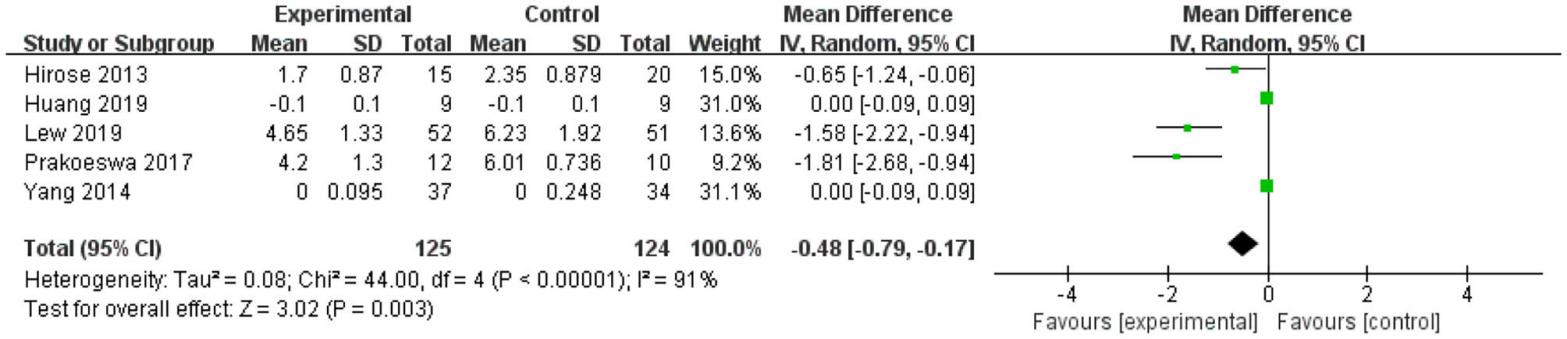
Forest plot of effect of *Lactobacillus plantarum* on IL-4. SD, standard deviation; Total, sample size of each group; Mean Difference, the mean value of the experimental group minus that of the control; 95% CI, 95% confidence intervals.

**Figure 3:**
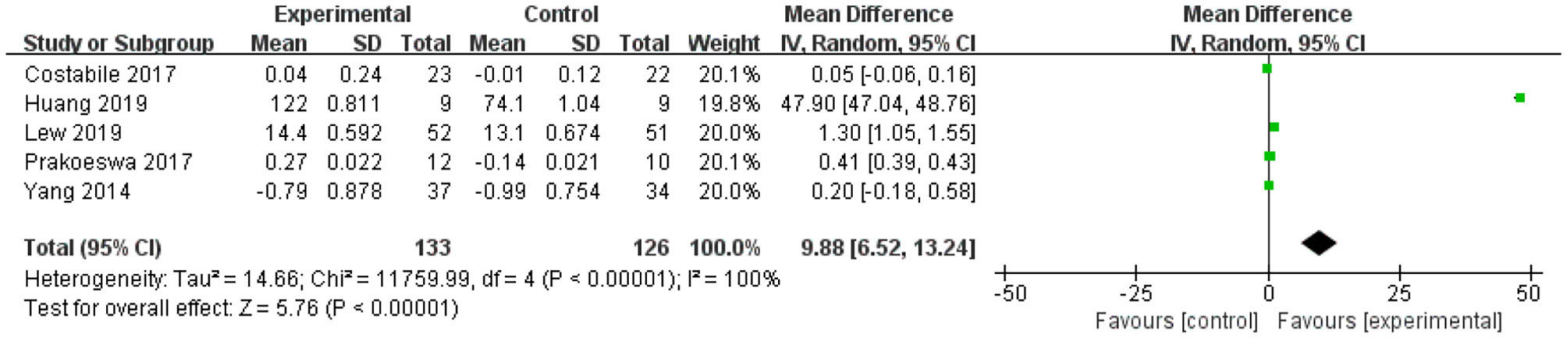
Forest plot of effect of *Lactobacillus plantarum* on IL-10. SD, standard deviation; Total, sample size of each group; Mean Difference, the mean value of the experimental group minus that of the control; 95% CI, 95% confidence intervals.

**Figure 4:**
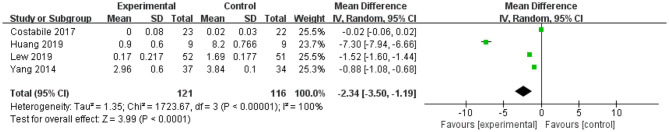
Forest plot of effect of *Lactobacillus plantarum* on TNF-α. SD, standard deviation; Total, sample size of each group; Mean Difference, the mean value of the experimental group minus that of the control; 95% CI, 95% confidence intervals.

**Figure 5:**
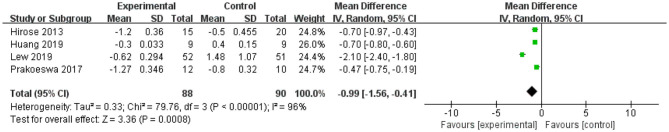
Forest plot of effect of *Lactobacillus plantarum* on IFN-γ. SD, standard deviation; Total, sample size of each group; Mean Difference, the mean value of the experimental group minus that of the control; 95% CI, 95% confidence intervals.

Review Manager 5.3 (Cochrane Collaboration Network, London, UK) was used to perform statistical analysis on four indicators: TNF-α (four studies), IL-4 (five studies), IFN-γ (four studies), and IL-10 (five studies). The mean difference and 95% confidence interval of these indicators are shown in Figures 2–**5**.

After intervention, the 95% CI of two of the five studies of IL-4 were 0 (the lower limit was <0 and the upper limit was >0) (Figure 2). The upper and lower limits of 95% CI of the other three studies were <0, indicating that these studies were statistically significant, and *L. plantarum* treatment was effective for IL-4. The mean difference of the total effect was −0.48 pg/mL (*p* < 0.05), its 95% CI was −0.79 to −0.17 pg/mL, indicating that *L. plantarum* could effectively decrease the level of IL-4 and modulate the host immunity (Figure 2).

For IL-10, the forest plot showed that the 95% CI of the first and last studies among the five included studies were 0 (the lower limit was <0 and the upper limit was >0), meaning that there was no statistical significance in this study and *L. plantarum* treatment was not effective in modulating the level of IL-10. The upper and lower limits of 95% CI of the other three studies were >0, indicating that these studies were statistically significant and that *L. plantarum* was effective in regulating the level of IL-10. The mean difference of the total effect was 9.88 pg/mL (*p* < 0.05), and the 95% CI was 6.52 to 13.2 pg/mL, suggesting the effectiveness of *L. plantarum* in enhancing the level of IL-10 and modulating the host immunity (Figure 3).

The forest plot of TNF-α (Figure 4) showed that the 95% CI in one of the four studies was 0 (the lower limit was <0 and the upper limit was >0), representing this study was not statistically significant for TFN-α. The lower and upper limits of the other three studies were all lower than 0, indicating that these studies were statistically significant, and *L. plantarum* treatment was effective in modulating the level of TNF-α. The mean difference of the total effect was −2.34 pg/mL (*p* < 0.05), and the 95% CI was −3.5 to −1.19 pg/mL, indicating that *L. plantarum* could effectively reduce the content of TNF-α and modulate the immune system (Figure 4).

The forest plot of IFN-γ revealed that the upper and lower limits of the 95% CI were lower than 0 in all four studies, indicating significant effects for IFN-γ. The *L. plantarum* treatment was effective for modulating the level of IFN-γ. The mean difference of the total effect was −0.99 pg/mL (*p* < 0.05), and the 95% CI was −1.56 to −0.41 pg/mL, suggesting that *L. plantarum* treatment could effectively reduce the content of IFN-γ to modulate the immune system (Figure 5).

In addition, we explored publication bias between included studies using funnel plots for all four immune parameters, and the results showed that slight publication bias existed across the 18 randomized controlled trials (Supplementary Figures 1–4).

## Discussion

This study performed a meta-analysis to evaluate the effect of *L. plantarum* in immune regulation. The results showed that *L. plantarum* significantly increased the level of IL-10 while significantly reduced the levels of IL-4, IFN-γ, and TNF-α (*p* < 0.05).

IL-4 is a T cell-derived growth factor for B cells. Over the last few decades, immunology studies have substantially expanded the knowledge about the cellular sources and functions of IL-4 (29). IL-4, as an anti-inflammatory cytokine, has been used in clinical biomaterials to promote wound healing after implantation surgery and reduce immune response (30). IL-4 mainly promotes the proliferation of CD8 + T cells, and endogenous IL-4 inhibits the secretion of IL-10 and promotes the release of TNF-α and GM-CSF (31). In a previous trial, *L. plantarum* IM76 significantly decreased IL-4 level in allergic mice, induced by house dust allergens (32). Similarly, the probiotic function of *L. plantarum* IS-10506, a probiotic strain isolated from the Indonesian fermented milk product *dadih*, could downregulate IL-4 in children with mild atopic dermatitis (17). Besides, the lysates of *L. plantarum* could inhibit IL-4 level in Nc/Nga mice and regulate inflammatory diseases like atopic dermatitis (33). The results of these studies are consistent with the current findings that the IL-4 level was significantly decreased by the consumption of *L. plantarum*. Interleukin-4 is a T cell-derived growth factor for B cells. In the last few decades, immunology studies have significantly expanded the knowledge about the cellular sources and functions of IL-4. Apart from T cells, IL-4 is also produced by myeloid cells, such as mast cells and basophils, and innate lymphocytes, such as NTK cells. It is a representative cytokine of the type 2 immune response, and it plays a direct role in modulating the host immunity (29).

The meta-analysis found that consuming *L. plantarum* enhanced IL-10 production. One study showed that *L. plantarum* 299 could regulate the ratio of Th1 and Th2 cells by modulating the production of IL-10 (34). Another study showed *L. plantarum* LS/07 could promote the IL-10 production in rats with colitis; and thus, *L. plantarum* LS/07 could be applicable in treating inflammation disease (35). Besides, administrating *L. plantarum* CIRM653 upregulated the IL-10 level in rats infected with *Klebsiella pneumonia* (36). As an anti-inflammatory factor, IL-10 is vital in protecting the host from tissue damage during acute phases of the immune response toward a pathogenic infection. Such regulatory mechanism helps maintain T cells homeostasis. In addition, IL-10 can be produced by almost all immune cells to autoregulate the functioning of these cells (37). IL-10 exerts immunosuppression through antigen presenting cells (APCs). IL-10 can significantly inhibit APCs, especially macrophages and dendritic cells (DCs). IL-10 can reduce the expression of major histocompatibility complex (MHC) class II molecules and co-stimulatory molecules (CD) 80 and CD86. Moreover, it can promote the expression of B7-H1 molecules and reduce the APC antigen presentation ability (38). Another important role of IL-10 is to inhibit the secretion of pro-inflammatory factors such as IL-1, IL-6, IL-12 and tumor necrosis factors by DCs and macrophages (39). In conclusion, IL-10 can act on many immune cell subsets and exert immunosuppression in various ways, which plays an important role in anti-inflammatory ailments.

The results showed that intake of *L. plantarum* significantly reduced the TNF-α level, which is in conformity with a previous study showing the supplementation of *L. plantarum* lowered the TNF-α concentration in male Wistar rats having metabolic syndrome (40). In addition, ingesting *L. plantarum* C4 could inhibit TNF-α production in male BALB/c mice after moderate physical exercise (41). Furthermore, one *L. plantarum* strain attenuated the TNF-α level in rats with non-alcoholic steatohepatitis (NASH) (42). TNF-α is a cytokine secreted mainly by macrophages, and it functions to modulate systemic inflammation by regulating immune cells. On the other hand, as an endogenous pyrogen, it can cause fever, apoptosis meanwhile prevent tumorigenesis and virus replication (43). In the inflammatory state, TNF-α can promote the expression of other inflammatory cytokines and aggravate inflammation. Moreover, it can also lead to the movement of neutrophils, interfere with the function of gastric mucosal endothelial cells and cause gastric mucosal damage (44). TNF receptor can transmit survival and death signals to cells, which plays an important role in cell proliferation, differentiation, apoptosis, regulation of immune response, and induction of inflammation (45).

The current meta-analysis found that consuming *L. plantarum* significantly reduced IFN-γ production in human subjects. Similarly, one published article found that administering *L. plantarum* KLDS1.038 downregulated IFN-γ secretion and promoted the recovery of mice by immunosuppression (46). The study of Lew and coworkers showed that *L. plantarum* P-8 could reduce the level of proinflammatory cytokines such as IFN-γ in stressed adults (27). Another study indicated that *L. plantarum* C4 could reduce IFN-γ production and avoid intestinal infections in mice through immunosuppression (47). Interferon-γ is a pro-inflammatory cytokine, which is mainly produced by activated NK cells and Th cells. The major biological effect of IFN-γ is immune-stimulation, inducing multiple antigen-presenting cells to express MHC-I/II molecules, activating macrophages and monocytes, enhancing lytic activity of immune cells, as well as enhancing the secretion of other cytokines such as IL-1, IL-6, IL-8, and TNF-α. Although IFN-γ serves as a pro-inflammatory cytokine and enhances the host immunity, which is desirable in many scenarios; however, its overshooting in some occasions might cause harmful tissue damages to the host. Thus, it would be useful to take advantage of the immunosuppression property of *L. plantarum* to protect from excessively strong immune responses in some cases (48).

Recently, several in depth studies have been conducted on the mechanisms of probiotics in immunoregulation (49, 50). However, specific underlying mechanisms of how *L. plantarum* interacts with the host are obscure.

Some studies have shown that *L. plantarum* strains exerted its immune regulatory function by competing with the pathogenic bacteria for limiting trophins, inhibiting the growth of pathogenic bacteria, regulating the intestinal microecology, and forming a biological barrier (51). Besides, *L. plantarum* produces lactic acid, diacetyl, and bacteriocin through metabolism, which could modulate the colonic environment and microbiome to improve its competitive advantages among other coexisting microflora (51, 52). Moreover, *L. plantarum* might interact with the host immunity via several mechanisms. Firstly, the immunomodulatory effect of *L. plantarum* could be attained via affecting the non-specific immune response and enhancing the viability of various types of immune cells, such as polymorphonuclear cells (mainly neutrophils) and cell involved in mononuclear phagocytosis (e.g., macrophages). These cells together with natural killer cells stimulate the secretion of mononuclear factors, such as lysosomal enzymes, and reactive oxygen species (53). The second mechanism is to affect the bacterial-specific immune responses, such as the increasing in the levels of IgA, IgG, and IgM in serum and the mucosal surface. Thus, augmenting the humoral immune function, promoting maturation and proliferation of B lymphocytes, T lymphocytes, and enhancing cellular immune response are some of the mechanisms of immunomodulation by *L. plantarum* (54). On the other hand, *L. plantarum* might reduce the production of pro-inflammation cytokines (such as TNF-α and IFN-γ) and induce peripheral tolerance via IL-10-induced antigen-specific T cell disability.

The meta-analysis showed that *L. plantarum* could significantly increase the level of IL-10 while decrease the levels of IL-4, TNF-α, and IFN-γ (*p* < 0.05). Both TNF-α and IFN-γ are pro-inflammatory cytokines. Many previous studies have drawn their conclusions on the effect of *L. plantarum* on the immune system by showing the up-regulation or down-regulation of a specific pro-inflammatory or anti-inflammatory factor, while the results found that *L. plantarum* could regulate the immunity via down-regulating multiple pro-inflammatory factors and up-regulating anti-inflammatory factors through a summary analysis of a large number of studies.

To minimize publication bias, this meta-analysis was performed objectively by two independent researchers. However, this study still has some unavoidable limitations. First, the heterogeneity of some indicators was significant, suggesting variations between studies. The discrepancies might be due to different types and doses of probiotics intervened in each study, as well as other factors such as diet, physical conditions, scale of study and so on. Second, each randomized controlled trials included in the current meta-analysis was performed in a different region, and the genetic background of subjects and their intrinsic microbiome might played a role in influencing the trial outcomes even if the same probiotics would have been applied. Third, the small sample size of some included studies might be affected the reliability and validity of the conclusions.

## Conclusions

We performed a meta-analysis on the effects of *L. plantarum* administration, mainly focusing on the immunoregulatory studies conducted in the last decade. The obtained results showed that *L. plantarum* significantly increased the level of IL-10, whereas, significantly reduced the level of IL-4, IFN-γ, and TNF-α (*p* < 0.05). Therefore, meta-analysis results suggested that *L. plantarum* could promote host immunity by co-regulating pro-inflammatory and anti-inflammatory cytokines.

## Data Availability Statement

The original contributions presented in the study are included in the article/Supplementary Material, further inquiries can be directed to the corresponding author.

## Author Contributions

WZhan designed the study. WZhao and CP retrieved references and extracted data of included references. WZhan and WZhao analyzed the data and discussed. WZhao and L-YK wrote the manuscript. HS edited and revised the final manuscript. WZhao, CP, HS, L-YK, and WZhan revised drafts and approved the final manuscript. All authors contributed to the article and approved the submitted version.

## Conflict of Interest

The authors declare that the research was conducted in the absence of any commercial or financial relationships that could be construed as a potential conflict of interest.
