# Supplementary material for: Meta-Analysis: Randomized Trials of Lactobacillus plantarum on Immune Regulation Over the Last Decades
Source: Front Immunol. 2021 Mar 22;12:643420. doi: 10.3389/fimmu.2021.643420 (PMC8019694; doi:10.3389/fimmu.2021.643420)
Supplement: Supplementary file 1 [file Data_Sheet_1.doc]

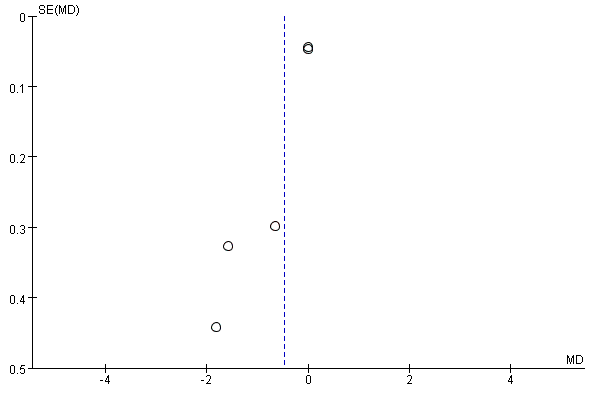


Figure S1 Funnel plot of effect of *Lactobacillus plantarum* on IL-4


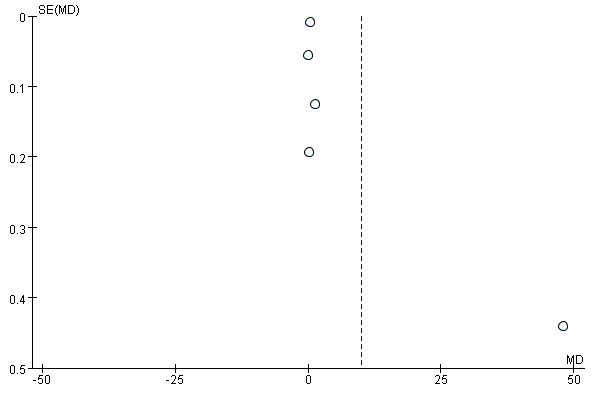


Figure S2 Funnel plot of effect of *Lactobacillus plantarum* on IL-10


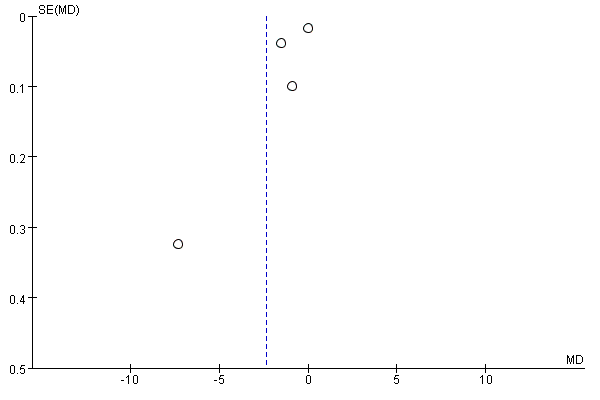


Figure S3 Funnel plot of effect of *Lactobacillus plantarum* on TNF-α


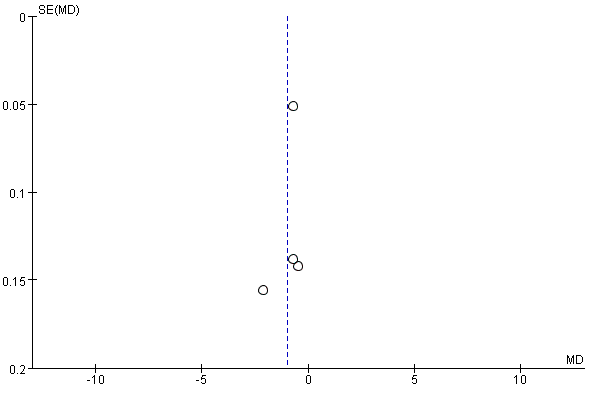


Figure S4 Funnel plot of effect of *Lactobacillus plantarum* on IFN-γ
